# Supplementary material for: Mucus polymer concentration and in vivo adaptation converge to define the antibiotic response of Pseudomonas aeruginosa during chronic lung infection
Source: mBio. 2024 Apr 23;15(6):e03451-23. doi: 10.1128/mbio.03451-23 (PMC11237767; doi:10.1128/mbio.03451-23)
Supplement: Supplemental figures — Fig. S1-S9. [file mbio.03451-23-s0001.pdf]

Supplemental Figures

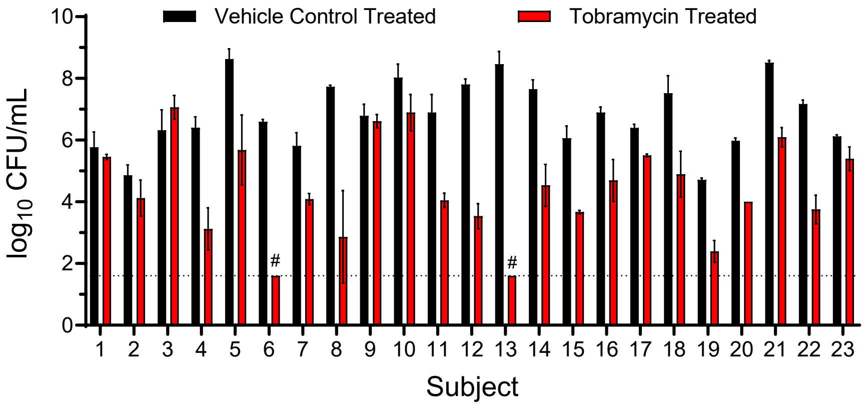

Figure S1. *Ex vivo* treated sputum CFU. CFU recovered on *Pseudomonas* isolation agar (PIA) from $n \geq 2$ sputum aliquots (mean ± SD) after 24 h incubation with vehicle control (PBS) or tobramycin (300 µg/mL). # indicates no recovered *P. aeruginosa* after antibiotic treatment (limit of detection, 4×10^1 CFU/mL).

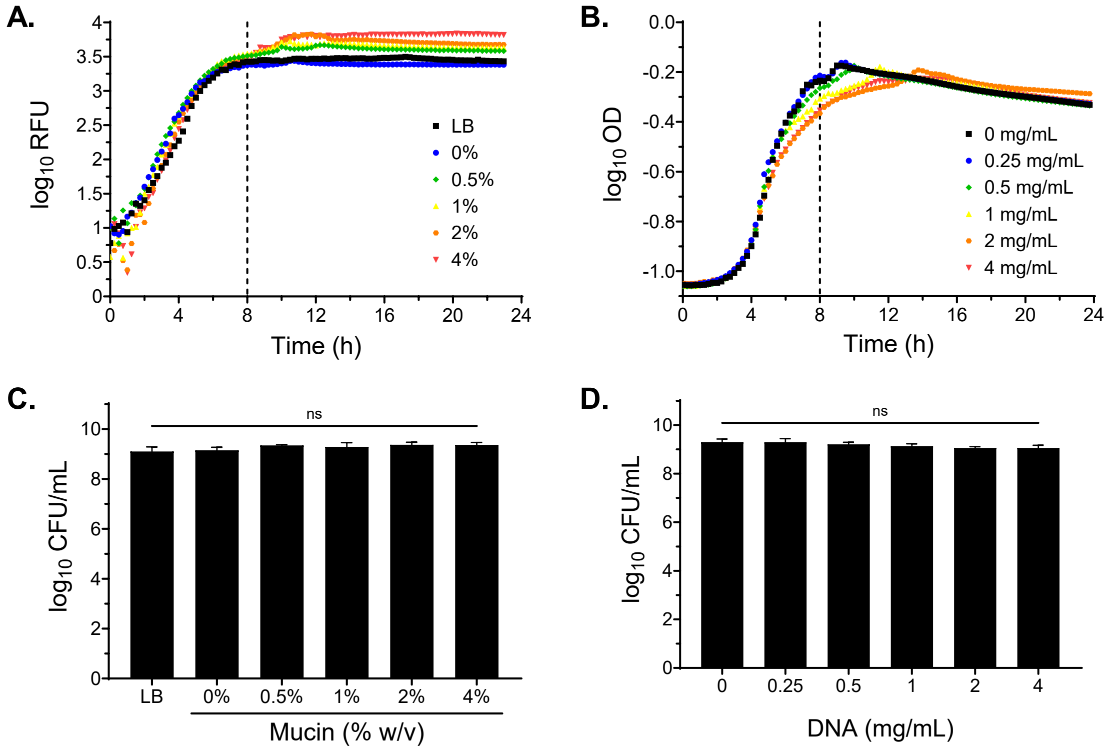

Figure S2. Mucin and eDNA do not affect growth kinetics of *P. aeruginosa*. (A) Growth curve of mPAO1 expressing *gfp*, in LB or SCFM2 with 0%, 0.5%, 1%, 2%, and 4% w/v mucin and no eDNA for 24 h. **(B)** WT mPAO1 growth curve in SCFM2 lacking mucin, with increasing concentrations of HMW eDNA. Fluorescence (483 / 535 nm) **(A)** or absorbance (600 nm) **(B)** measurements were taken every 15 min using a Tecan Infinite 200 PRO plate reader (Tecan Group Ltd., AG, Switzerland). Data are representative of the mean from *n* = 3 biological replicates in technical duplicate. (**C and D**) CFU of mPAO1 after 8 h of growth in SCFM2 with (C) increasing mucin or (D) increasing eDNA concentration. Statistical analysis performed by one-way ANOVA with Tukey's multiple comparison correction. ns indicates not significant (*p*-value > 0.05).

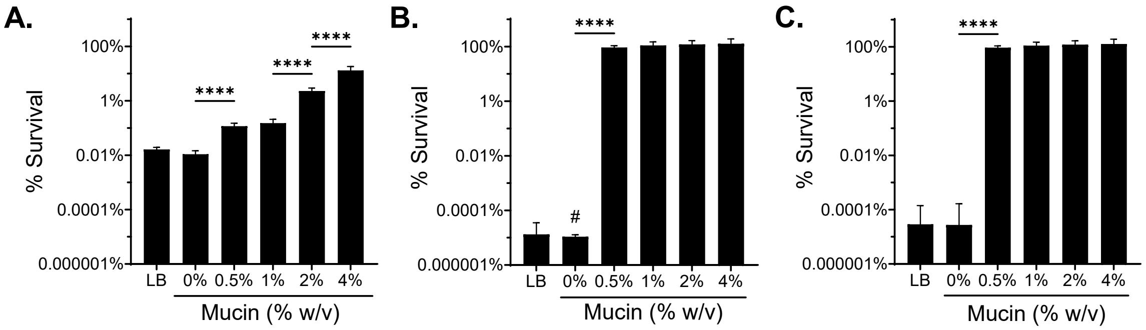

Figure S3. ***P. aeruginosa*** **exhibits mucin concentration-dependent tolerance to multiple antibiotic classes.** Survival of mPAO1 cultured in LB and SCFM2 media with increasing % w/v mucin and treated with **(A)** 300 µg/mL ciprofloxacin, **(B)** 40 µg/mL colistin, or **(C)** 40 µg/mL polymyxin B for 24 h. Mean ± SD of $n \geq 3$ biological replicates. Statistical differences were analyzed using one-way ANOVA with Tukey's multiple comparison correction. ****p<0.0001. # indicates no recovered *P. aeruginosa* after antibiotic treatment (limit of detection, 10^2 CFU/mL).

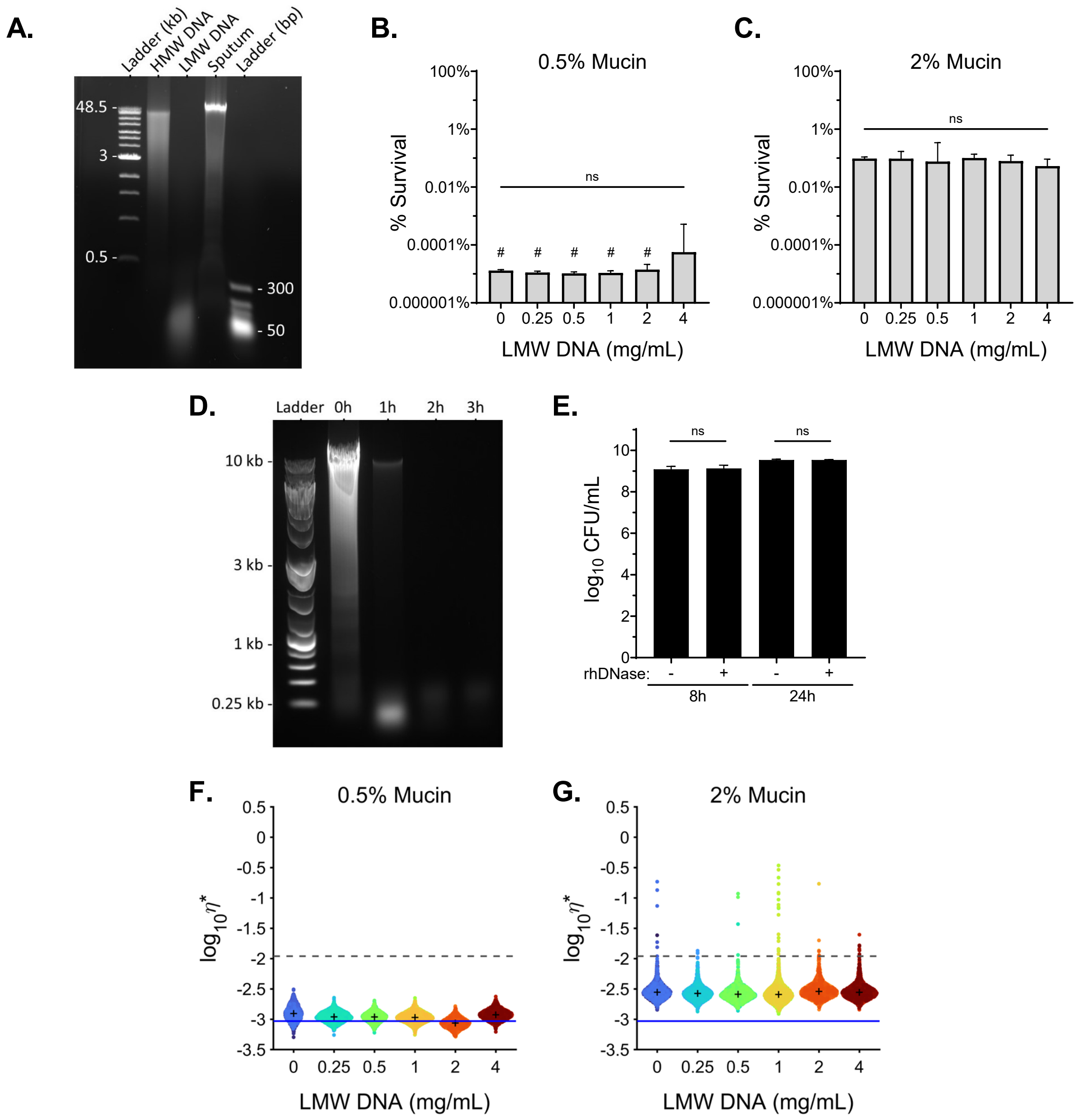

Figure S4. High molecular weight eDNA is necessary to impart tobramycin tolerance. (A) DNA gel electrophoresis of salmon sperm DNA from different commercial sources compared to a representative sputum from a CF subject. **(B-C)** % survival of mPAO1 after treatment with 300 µg/mL tobramycin in SCFM2 with **(B)** 0.5% or **(C)** 2% w/v mucin and increasing concentrations of LMW eDNA. Statistical differences were determined by one-way ANOVA. **(D)** Gel electrophoresis of SCFM2 containing 2% mucin and 1 mg/mL HMW DNA treated with rhDNase (3 µg/mL) for 0, 1, 2 or 3 h. **(E)** Effect of rhDNase (3 µg/mL) on the growth of mPAO1 in 2% mucin SCFM2 after

8 and 24 h. Mean ± SD of $n \geq 3$ biological replicates. ns indicates not significant (p-value > 0.05). Probability distribution of each microbead η^* for increasing LMW eDNA concentrations in **(F)** 0.5% or **(G)** 2% w/v mucin SCFM2. Data are representative of $n \geq 3$ technical replicates. The viscosity of water is indicated by a solid blue line and the complex viscosity at which mucus entangles and becomes gel-like (42) is indicated by a gray dashed line. + indicates the median complex viscosity for each sample.

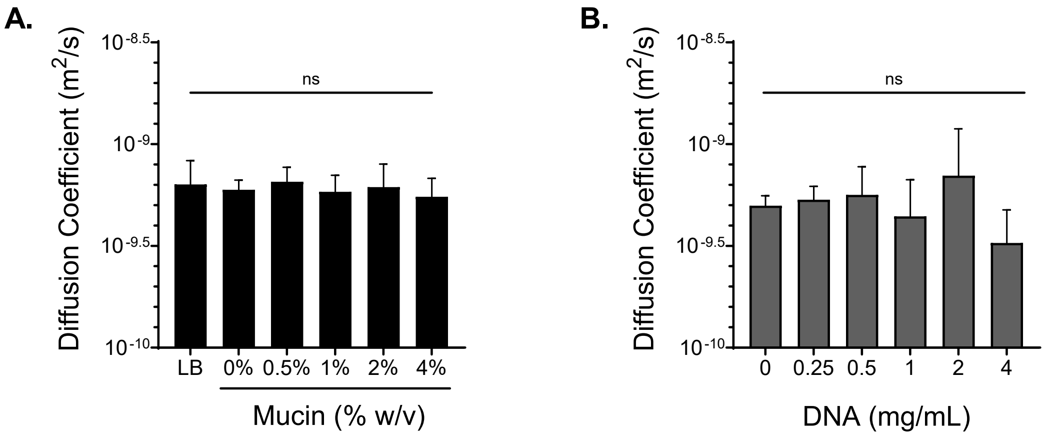

Figure S5. Antibiotic diffusion was not affected by polymer concentration. Diffusion rate of Texas Red conjugated tobramycin through **(A)** LB and SCFM2 with increasing mucin concentrations and **(B)** 0.5% w/v mucin SCFM2 with increasing concentrations of eDNA. Data are presented as the mean ± SD of n = 3 replicates. Statistical analysis was performed by one-way ANOVA. ns indicates not significant (p-value > 0.05).

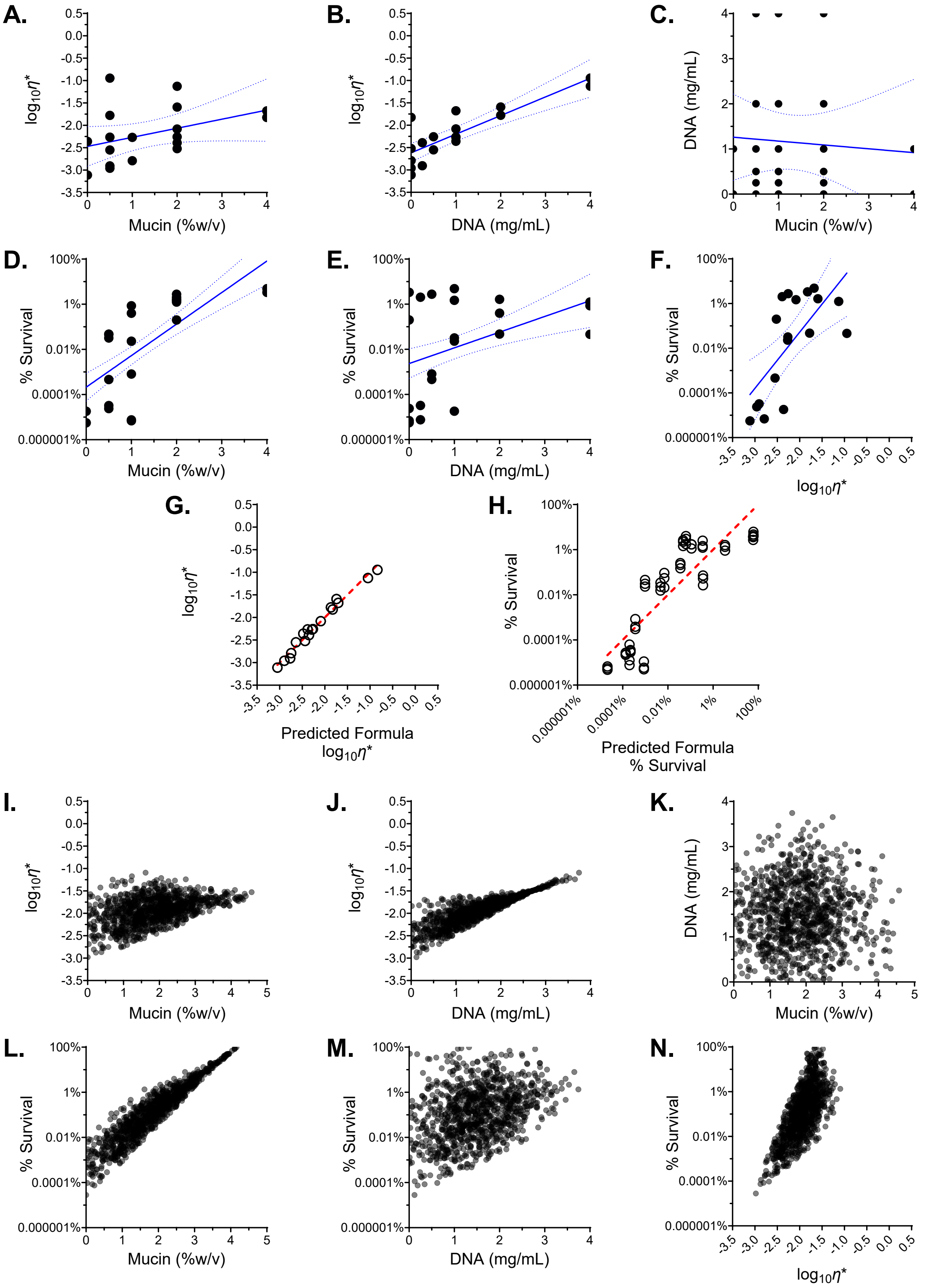

Figure S6. PLS regression model and simulation. (A-F) Linear trend and 95% confidence intervals of the relationship between the outcome variables (tobramycin survival and

viscoelasticity) and input variables (mucin and eDNA concentration). **(G-H)** Three-factor model performance comparing the actual versus model predictions for **(G)** viscoelasticity and **(H)** antibiotic survival. **(I-N)** Individual biplot of 1000 simulated runs through the interactive model demonstrating the relationship between the outcome variables (tobramycin survival and viscoelasticity) and input variables (mucin and eDNA concentration).

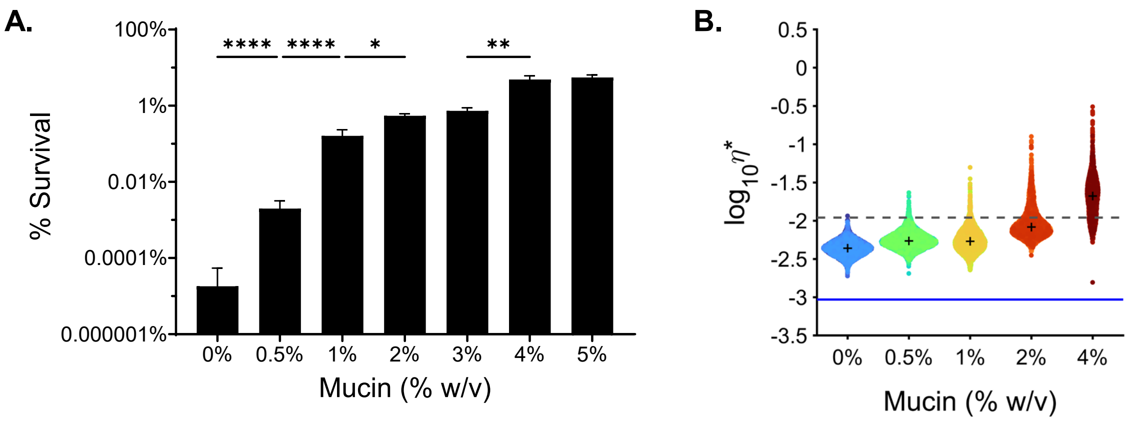

Figure S7. Antibiotic survival and complex viscosity of final media composition. (A) Tobramycin survival of mPAO1, and **(B)** complex viscosity of SCFM2 containing 1 mg/mL HMW eDNA and increasing % w/v mucin. Statistical differences were determined using one-way ANOVA with Tukey's multiple comparison correction. *p<0.05, **p<0.01, ****p<0.0001. Data represent the mean ± SD of $n \geq 3$ biological replicates. The viscosity of water is indicated by the solid blue line, and the complex viscosity at which mucus entangles and becomes gel-like (42) is indicated by the dashed gray line. + indicates the median for each sample.

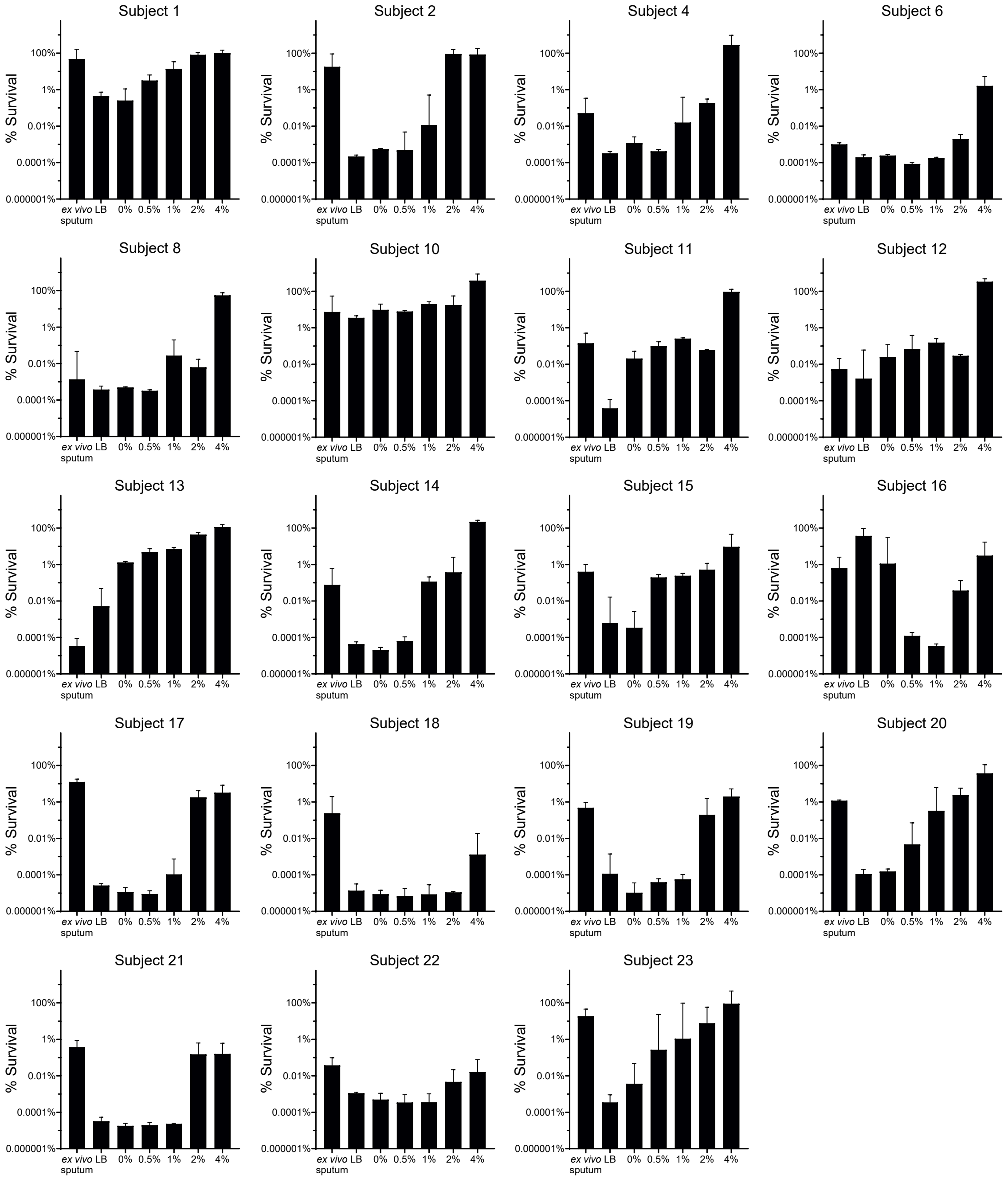

Figure S8. ***In vitro* mucin concentration-dependent antibiotic survival of clinical populations.** Percent survival of *ex vivo* treated sputum and *P. aeruginosa* populations recovered from each subject mock treated sputum evaluated *in vitro* for tobramycin tolerance in LB and SCFM2 containing 1 mg/mL HMW eDNA with increasing %w/v mucin. Hyperresistant sputum populations (MIC ≥ 80) were excluded. Data are representative of *n* = 3 independent replicates

and plotted as mean ± SD. # indicates no recovered *P. aeruginosa* after tobramycin treatment (limit of detection, 10^2 CFU/mL).

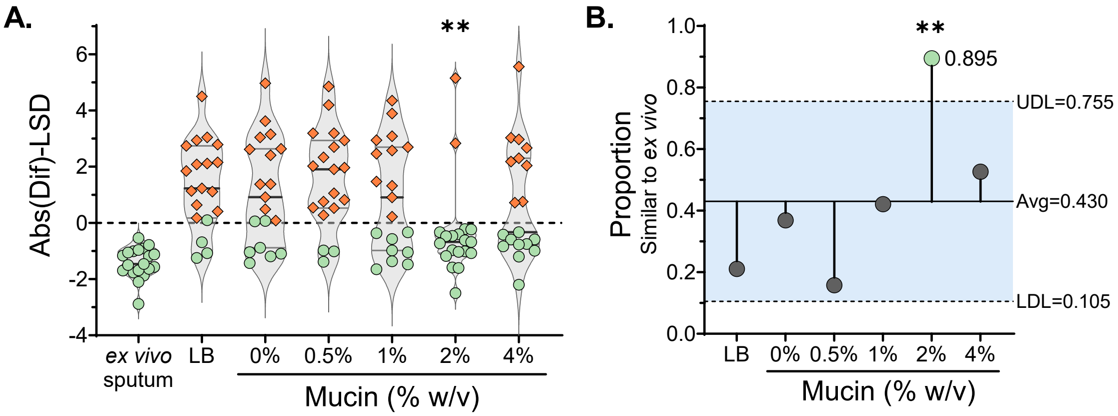

Figure S9. *In vitro* 2% mucin SCFM2 recapitulates *ex vivo* sputum antibiotic efficacy. (A) Comparison of the absolute difference between sample means minus Fisher's least significant difference (Abs (Dif) - LSD). Values > 0 (orange circles) were considered different from *ex vivo* conditions at Dunnett's post-test *p*-values < 0.05. **(B)** Proportion of *in vitro* cultures similar to *ex vivo* sputum tobramycin survival. Conditions that were disproportionate compared to the overall mean (43%) were identified via analysis of means for proportions (α = 0.01). The 2% mucin condition was the only condition above the upper decision line (UDL), indicating a difference from the mean across all conditions. $^{**}p<0.01$.
